# FoxC1-Induced Vascular Niche Improves Survival and Myocardial Repair of Mesenchymal Stem Cells in Infarcted Hearts

**DOI:** 10.1155/2020/7865395

**Published:** 2020-07-04

**Authors:** Lan Zhao, Rui Zhang, Feng Su, Libing Dai, Jiahong Wang, Jin Cui, Weiguang Huang, Shaoheng Zhang

**Affiliations:** ^1^Department of Cardiology, GuangZhou Red Cross Hospital Medical College of Ji-Nan University, 396 Tongfuzhong Road, Haizhu District, Guangzhou 510220, China; ^2^Department of Cardiology, Dahua Hospital, 901 Laohumin Road, Xuhui District, Shanghai 200237, China; ^3^Department of Cardiology, Yangpu Hospital, Tongji University School of Medicine, 450 Tengyue Road, Shanghai 200090, China

## Abstract

**Aims:**

Forkhead box C1 (FoxC1) is essential for maintaining the hair follicle stem cell niche. The role of FoxC1 in maintaining mesenchymal stem cell (MSC) niches after myocardial infarction (MI) has not been directly determined to date. In this study, we determined to explore the possible roles and mechanisms of FoxC1 on MSC survival and function in the ischemic niche.

**Methods and Results:**

MI model was established in this study, and expression level of FoxC1 was overexpressed or knocked down through efficient delivery of FoxC1 transfection or *si*FoxC1. Fifteen days later, the animals were allocated randomly to receive phosphate-buffered saline (PBS) injection or MSC transplantation. We identified FoxC1 as a key regulator of maintaining the vascular niche in the infarcted hearts (IHs) by driving proangiogenic and anti-inflammatory cytokines while repressing inflammatory and fibrotic factor expression. This vascular niche improved MSC survival and capacity in the IHs. Importantly, FoxC1 interacted with MSCs and was required for vessel specification and differentiation of engrafted MSCs in the ischemic niches, promoting myocardial repair. Inhibiting FoxC1 abolished these effects.

**Conclusion:**

These results definitively implicate FoxC1 signaling in maintaining ischemic vascular niche, which may be helpful in myocardial repair induced by MSC therapy.

## 1. Introduction

Mesenchymal stem cells (MSCs) are a promising cell type being evaluated for patients with heart failure (HF) secondary to ischemic cardiomyopathy [1]. However, the therapeutic potency of transplanted MSCs appears to be limited by low rates of engraftment, survival, and differentiation [2]: the percentage of transplanted MSCs in hearts declined from 34–80% immediately after administration to just 0.3–3.5% after 6 weeks [3]. It is not entirely clear which variables might influence outcome, where ischemic/necrotic tissue may create an entirely different niche to dictate cell fate. Our previous study suggested that inflammation and insufficient vascular supply resulting from ischemia and the loss of angiogenic factors after myocardial infarction (MI) limit transplanted stem cell function and numbers [4]. Therefore, exploration of the mechanisms on how MSC survival niches can be improved may be required to maximize their beneficial effects on myocardial repair.

Previous evidence has suggested that forkhead box C1 (FoxC1) is essential for maintaining the hair follicle stem cell niche and governing stem cells to preserve long-term tissue-regenerating potential [5]. FoxC1 is a member of the subfamily FoxC of the forkhead/winged-helix transcription factor (Fox) family, which plays a pivotal role in regulating heart development and stem/progenitor cell niche formation [5–7]. However, the FoxC1 network regulatory system is very complex in the niches that maintain stem cell survival and function. The molecular mechanisms of how FoxC1 affects stem cell function and survival in the ischemic niche of infarcted hearts (IHs) are poorly understood.

Some have suggested that FoxC1 can control corneal vascular growth by regulating proangiogenic factors [8]. We recently identified a host vascular niche essential for delaying apoptosis and enhancing the regenerative properties of stem cells engrafted into IHs, which is coordinated by a series of powerful angiogenic factors that include vascular endothelial growth factor (VEGF), angiopoietin-1 (Ang-1), and basic fibroblast growth factor (bFGF) [4, 9]. Here, we explored the possibility of FoxC1 as a key component in maintaining the vascular niche of IHs and determining its effects on survival and myocardial repair of MSCs in the IHs.

## 2. Materials and Methods

The Supplementary Information section online contains an expanded Materials and Methods section with details on animal allocation, animal model, study design, MSC collection and transplantion, echocardioraphy, histology, immunofluorescence, ELISA, qRT-PCR, immunoblotting, and statistical analysis.

### 2.1. Construction of Plasmids and Preparation of Recombinant Adeno-Associated Virus (rAAV)

To manipulate the expression of FoxC1, the rAAV systems were employed. The oligonucleotides and their complementary ones were synthesized by USEN Tech (Shanghai, China), then annealed and ligated into the vectors. For the expression of FoxC1, the full-length sequence of its protein coding sequence was amplified by PCR using the primers and then ligated into the vectors. *ad*FoxC1 or FoxC1 siRNAs and control siRNA duplexes were transfected together with the rAAV plasmid vector into IHs, as described previously [10, 11].

### 2.2. MI Model and FoxC1-Induced Niches

Inbred Lewis rats were used. The Animal Care and Use Committee of Yangpu Hospital, Tongji University School of Medicine approved all animal experiments, which were in compliance with the Guide for the Care and Use of Laboratory Animals published by the National Academy Press. IHs were induced in the rats by ligating the left anterior descending coronary artery. Animals with an ejection fraction (EF) < 70% and fractional shortening (FS) < 35% evaluated by echocardiography after induction of IHs were selected. To investigate the effects of FoxC1 on the ischemic niche, the animals were randomly divided into three groups corresponding to Foxc1 transfection status: knockdown of FoxC1 by transfecting the IHs with vectors encoding FoxC1 siRNA (*si*FoxC1 group), no intervention, and overexpression of FoxC1 by transfecting the IHs with vectors encoding FoxC1 (*ad*FoxC1 group). No-intervention rats were transfected with control vectors (CON). After 15 days postinduction, ten animals in each group were sacrificed to exactly valuate the FoxC1-induced vascular environment at the tissue, cellular, and molecular levels. Meanwhile, other animals received either vehicle (phosphate-buffered saline [PBS]) or MSC injection.

### 2.3. Enzyme-Linked Immunosorbent Assays (ELISA)

The levels of FoxC1, Ang-1, bFGF, and VEGF in the supernatant of heart tissues were measured by ELISA using a DuoSet methodology (R&D Systems; Minneapolis, MN).

### 2.4. Isolation, Culture, and FACS of MSCs

BM-derived mononuclear cells (BM-MNCs) were isolated and collected from the bilateral femora and tibiae of the rats. MSCs were isolated from rat BM-MNCs and cultured via the adherent culture method as we previously described [12, 13]. The characters of the MSCs were confirmed with antibodies against CD34, CD44, CD71, CD90, CD147, SH2, SH3, CD45, and CD133 by fluorescence activating cell sorter (FACS). MSCs were analyzed by FACS. MSC expression of the MSC markers SH2, SH3, CD44, CD71, CD90, and CD147 showed positivity of 90.6%, 89.4%, 97.8%, 86.5%, 99.4%, and 91.33%, respectively; and for hematopoietic markers, CD34, CD45, and CD133 showed negativity of 2.74%, 3.56%, and 4.98%, respectively, which indicates the purity of MSCs (Fig. S3).

### 2.5. EGFP Labeling

To label the transfected cells genetically, we constructed a lentivirus vector inserted with an enhanced green fluorescent protein (EGFP) cDNA as described previously [9] and transfected it into some MSCs before transplantation. More than 50% of MSCs were EGFP-positive, as determined by flow cytometry.

### 2.6. Cell Therapy and Group

At 15 days postoperation, the animals were then randomized to receive saline injection or cell therapy. Twenty animals were studied in each subgroup. Cyclosporin A (Novartis Pharma) was administered (5 mg/kg, IH) starting at day 0 and daily thereafter until the end of each experiment.

### 2.7. Follow-Up

All rats underwent echocardiography to evaluate cardiac functions before transplantation and at 30 days postcell therapy and were closely observed up to death to evaluate their survival curves.

### 2.8. Echocardiography

Thirty days later, cardiac functions were evaluated by echocardiographic assessments of LV fractional shortening (LVFS), left ventricular end-diastolic volume, internal diameter, anterior wall thickness, and posterior wall thickness at the diastolic phase (LVEDV, LVEDD, LVAWd, and LVPWd, respectively), and the structural benefits of therapy were evaluated by measuring the LV infarct size as determined by echocardiography.

### 2.9. MPO and ROS Detection

Myeloperoxidase (MPO) and reaction oxygen species (ROS) in heart tissues were detected using kits according to the manufacturer's instructions.

### 2.10. Histology

The infarct size was determined by calculating the percentage of the infarcted area against the whole LV area using Scion ImageJ. The peri-infarct regions from the MI model rats and cell therapy rats were embedded in paraffin, sectioned, and stained with triphenyltetrazolium chloride (TTC), hematoxylin and eosin (H&E), and Masson's trichrome or by immunohistochemistry or immunofluorescence.

### 2.11. qRT-PCR and Immunoblotting

Peri-infarct myocardial tissues were harvested and pulverized to extract RNA or protein for qRT-PCR or immunoblotting, respectively.

### 2.12. Analysis of Cell Proliferation

Cell proliferation was assessed by fluorescence staining for the proliferation marker Ki67 using FACS or immunofluorescence.

### 2.13. Statistical Analysis

The results are expressed as the mean ± SEM and were tested for significance using multiple-comparison analysis of variance. Chi-square analysis was used to compare survival rates between groups. A *p* value of <0.05 was considered statistically significant.

### 2.14. Online Data: Supplementary Tables and Figures

Supplementary Table S1 and Table S2 list the qRT-PCR primers and the antibodies for western blotting, immunofluorescence, and immunohistochemistry, respectively. Supplementary Fig. S1 in the online-only data presents the treatment flowchart and the cell and animal groups. Supplementary Fig. S2 in the online-only data exhibits the expression time curve of FoxC1-related vascular growth factors. Supplementary Fig. S3 displays the surface marker expression of rat MSCs. Supplementary Fig. S4 reveals local engraftments and myocardiogenesis of EGFP^+^MSCs in the IHs. Supplementary Fig. S5 in the online-only data shows the model of FoxC1 mediation of myocardial repair of engrafted MSCs in vascular niches.

## 3. Results

### 3.1. FoxC1-Induced Host Vascular Niche in Infarcted Hearts

**Figure 1:**
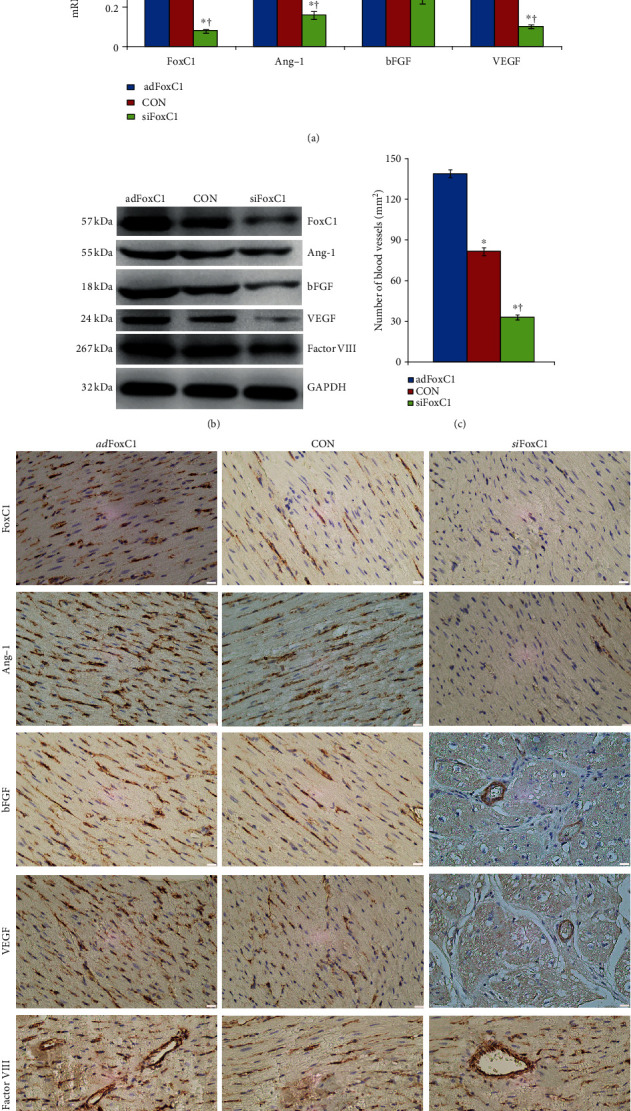
FoxC1-induced vascular niche at 15 days after MI. MI rat hearts were transfected with Foxc1 vector (*ad*Foxc1), FoxC1 siRNAs (*si*FoxC1), or control siRNA duplexes (CON) immediately after MI induction. (a) Real-time qRT-PCR quantitative analysis data of FoxC1, Ang-1, bFGF, and VEGF mRNA in the *ad*FoxC1 group, CON group, and *si*FoxC1 group 15 d post-MI. (b) Immunoblotting analyses show the representative expression of FoxC1 and its downstream growth factors. (c) Quantification data of vessel numbers in these groups. (d) Immunohistochemical staining showing positive staining (brown) of FoxC1, Ang-1, bFGF, and VEGF at the peri-infarct area. Representative slides show factor VIII^+^ ECs in the peri-infarct regions from the *ad*FoxC1 group, CON group, and *si*FoxC1 group. Scale bars = 50 *μ*m. All data are the means ± SEM. *p* < 0.05: ^∗^vs. *ad*FoxC1 group, ^†^vs. CON group (*n* = 20 per group); Student's *t*-test.

FoxC1 appears to control pathological angiogenesis by regulating VEGF signaling [11]. Previously, we suggested that the existence of an intrinsic vascular niche appears more beneficial to cardiac repair induced by stem cell therapy after MI [4, 9]. We hypothesized that FoxC1 may play a role in maintaining this vascular niche and tested this hypothesis using an IH rat model by FoxC1 overexpression or siRNA inhibition. First, we examined the expression time curve of FoxC1-related vascular growth factors in the *ad*FoxC1-transfected IHs, the CON IHs, and the *si*FoxC1-transfected IHs. ELISA showed that FoxC1 expression gradually increased over time, peaked at 15 days after infarction, and then decreased. Moreover, FoxC1 transfection caused a further increase of its expression in the *ad*FoxC1 IHs at each time point and maintained its persistent increase at 30 d post-MI. However, FoxC1 knockdown weakened this increase (Fig. S2a). The expressions of vascular growth factors including Ang-1, bFGF, and VEGF in the IHs were parallel to FoxC1 expression, and their expression patterns were seen with the greatest significance in the *ad*FoxC1 day 15 IHs (Fig. S2b, 2c, and 2d), which was consistent with our previous findings demonstrating that a best vascular niche was shown in the infarcted hearts with collateral vessels at 2 weeks post-MI [4]. Therefore, we concluded that FoxC1-mediated angiogenic cytokines were at their highest levels at 15 d post-MI, which reached the best point of vascular niches for MSC therapy. We hereby chose 15 d post-MI as the time point of cell therapy to investigate the effect of various niches in an infarcted rat model. Furthermore, we performed qRT-PCR, western blot, and immunohistochemistry to explore the consequences of FoxC1 overexpression by evaluating the changes in FoxC1-related vascular growth factors and blood vessel density around the peri-infarct myocardial tissues from MI rats 15 days after *ad*Foxc1 transfection. The *ad*FoxC1 group had considerably higher FoxC1 mRNA and protein expression levels than the CON group. When the MI hearts were knocked down by *si*FoxC1, FoxC1expression levels were greatly decreased in the *si*FoxC1 group (Figures 1(a) and 1(b)). These expression phenotype changes were reflected in the expressions of vascular growth factors: the *ad*FoxC1 group had higher Ang-1, bFGF, and VEGF mRNA and protein levels than the CON groups; FoxC1 inhibition greatly decreased the expression levels of these proangiogenic cytokines in the *si*FoxC1 group (Figures 1(a) and 1(b)). The immunohistochemistry results showed that the *ad*FoxC1 group had the most positive expression particles for FoxC1, Ang-1, bFGF, and VEGF, followed by the CON group; the least was found in the *si*FoxC1 group (Figure 1(d)). To test the effect of FoxC1 on ischemia-induced host intrinsic angiogenesis, we investigated factor VIII expression levels via western blotting and immunohistochemical staining in the peri-infarct regions. Factor VIII expression was highest in the *ad*FoxC1 group, followed by that in the CON group; the expression was lowest in the *si*FoxC1 group. The greatest vessel density showed the same trend in these groups (Figures 1(c) and 1(d)). Therefore, *ad*Foxc1 transfection can contribute to the establishment of a vascular niche in the IHs. Next, we determined whether the FoxC1-induced niche could benefit from the survival and myocardial repair of MSCs in the IHs.

### 3.2. FoxC1-Induced Niche Augments Myocardial Repair of MSC Therapy

**Figure 2:**
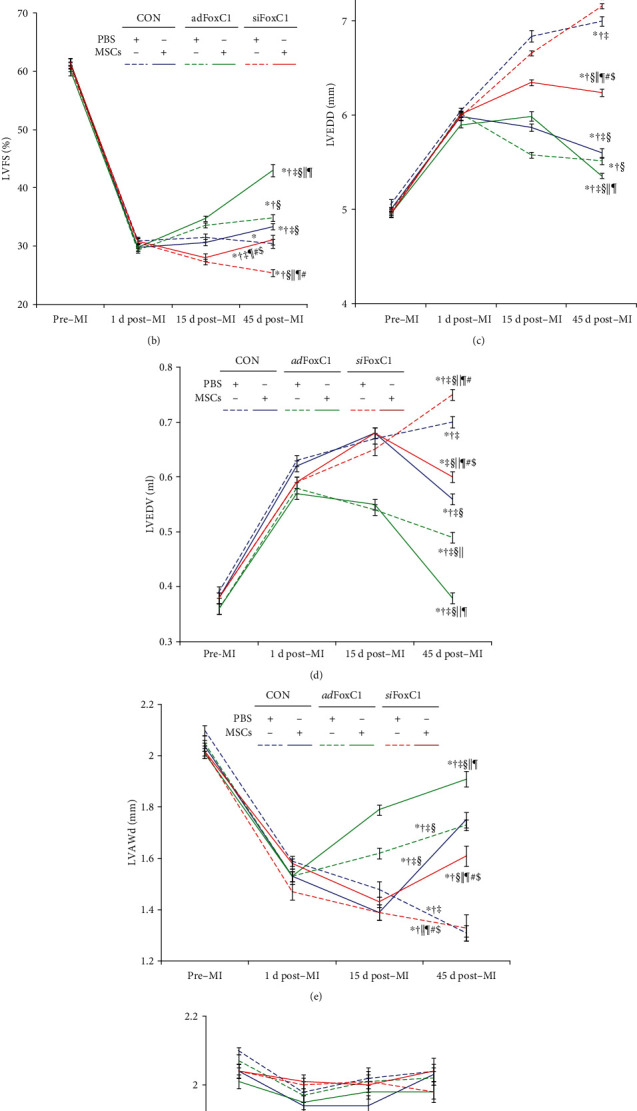
FoxC1-induced niche augments the effects of MSC therapy. (a) Kaplan–Meier survival rates. (b–f) Echocardiography of LVFS (b), LVEDD (c), LVEDV (d), LVAWd (e), and LVPWd (f) before MI (pre-MI), 1 d after MI (1 d post-MI), 15 d after MI (before cell therapy, 15 d post-MI), and 45 d after MI (30 d after cell therapy, 45 d post-MI). FoxC1 transfection plus MSC transplantation had higher survival rates and significant improvement in cardiac function and structural remodeling. However, this was cancelled in the *si*FoxC1 group. All graphical data are the mean ± SEM. *p* < 0.05: ^∗^vs. pre-MI, ^†^vs. 1 d post-MI, ^‡^vs. 15 d post-MI, ^§^vs. CON+PBS, ^║^vs. CON+MSCs, ^¶^vs. *ad*FoxC1+PBS, ^#^vs. *ad*FoxC1+MSCs, and ^$^vs. *si*FoxC1+PBS. Pre-MI, 1 d post-MI, and 15 d post-MI, *n* = 20 per group. At 45 d post-MI, CON IHs: PBS, *n* = 11, and MSCs, *n* = 13; *ad*FoxC1 IHs: PBS, *n* = 15, and MSCs, *n* = 18; *si*FoxC1 IHs: PBS, *n* = 9, and MSCs, *n* = 10; Student's *t*-test.

MI rats 15 days after depletion or overexpression of FoxC1 treated with *si*FoxC1 or *ad*FoxC1 randomly received PBS injection or MSC implantation. The 120 animals were randomly divided into six groups and underwent serial echocardiography studies (Fig. S1). Thereafter, all animals were followed up for 30 days, during which 44 rats died. The surviving 76 rats were then sacrificed and subjected to pathology and molecular biology tests. After 30 days, the Kaplan–Meier survival analysis showed a higher survival rate in the *ad*FoxC1 IHs receiving MSC transplantation than in other rats (90% in the *ad*FoxC1 IHs receiving MSCs versus 55% in the CON IHs receiving PBS, *p* = 0.019; versus 65% in the CON IHs receiving MSCs, *p* = 0.048; versus 45% in the *si*FoxC1 IHs receiving PBS, *p* = 0.004; versus 55% in the *si*FoxC1 IHs receiving MSCs, *p* = 0.007). However, no significant benefit in survival rate was seen in the animals receiving FoxC1 transfection alone or MSC transplantation alone, and this effect was canceled in the *si*FoxC1 IHs (Figure 2(a)).

Echocardiographic studies showed that on day 1 postinfarct, animals in all six groups developed typical changes of acute heart failure and LV early remodeling, in comparison with data obtained at the baseline levels. These changes included decreased cardiac function index LVFS, dilated LVEDD and LVEDV, and thinning of LVAWd (Figures 2(b)–2(e)). Although transfection of FoxC1 transiently improved LVFS, LVEDD, LVEDV, and LVAWd in the *ad*FoxC1 IHs at 15 d post-MI, these benefits in LVFS and LVEDD were not sustained in the *ad*FoxC1 IHs receiving PBS at 45 d post-MI. At 45 days, the rats with the *ad*FoxC1 IHs that had received MSCs exhibited sustained improvement in LVFS, LVEDD, and LVEDV. These beneficial effects were eliminated in the *si*FoxC1 IHs. The indices in the CON and the *si*FoxC1 IHs receiving PBS indicated sustained exacerbation with decrease of LVEF, dilation of LVEDD and LVEDV, and thinning of LVAWd. There was no significant difference in LVPWd between the six groups (Figure 2(f)*p* > 0.05). Taken together, these data suggest that the overexpression of FoxC1 in the ischemic niche improves the effects of MSC therapy in restoring cardiac function and structure.

### 3.3. FoxC1 Improved the Pathological Microenvironment of Engrafted MSCs

**Figure 3:**
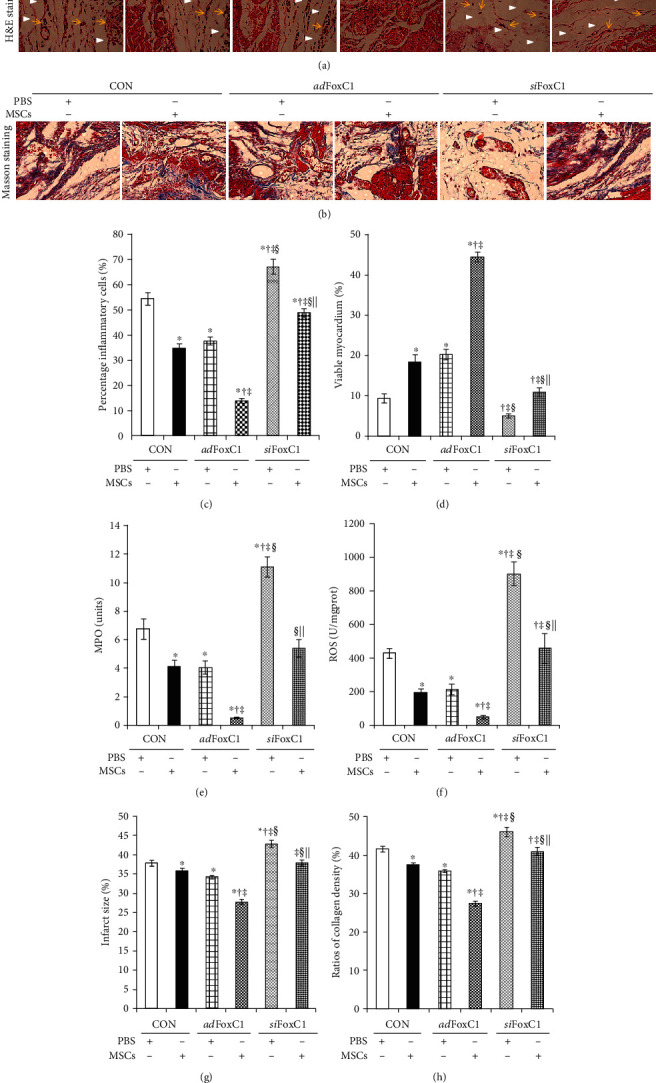
FoxC1 enhances MSC-mediated amelioration of MI pathology. (a, b) Representative high-magnification images of H&E staining (a) (yellow arrows, inflammatory cell infiltration; white arrowheads, myocyte necrosis) and Masson's trichrome staining (b) (none of the infarcted myocardium was stained red; pale region, infarcted myocardium; bright red, viable myocardium; bright blue, fibrosis) at 30 days post-MI. Scale bars = 50 *μ*m. (c–h) Quantitative analysis of inflammatory cells (c), viable myocardium (d), MPO (e), ROS (f), infarct size (g), and collagen content (h) 30 days after cell therapy (45 d post-MI). All graphical data are the mean ± SEM. ^∗^*p* < 0.05 vs. CON+PBS, ^†^*p* < 0.05 vs. CON+MSCs, ^‡^*p* < 0.05 vs. *ad*FoxC1+PBS, ^§^*p* < 0.05 vs. *ad*FoxC1+MSCs, and ^||^*p* < 0.05 vs. *si*FoxC1+PBS (CON IHs: PBS, *n* = 6, and MSCs, *n* = 7; *ad*Foxc1: PBS, *n* = 10, and MSC*s*, *n* = 13; *si*FoxC1: PBS *n* = 4, and MSCs, *n* = 6); Student's *t*-test. FoxC1 enhanced the reduction of myocardial inflammation and collagen and increased angiogenesis induced by MSC therapy, suggesting that FoxC1 may contribute to the amelioration of MI pathology in cooperation with MSC therapy.

To examine the potential effect of FoxC1 on myocardial remodeling, we evaluated myocardial inflammation, infarct, and fibrosis using H&E, TTC, and Masson's trichrome staining. H&E staining showed that, in the CON group, compared with PBS injection, MSC therapy did significantly reduce MI-induced significant inflammation, including neutrophil infiltration, myocyte loss, and bleeding. MSC transplantation into the *ad*FoxC1 IHs caused the greatest reduction of myocardial inflammation. However, this reduction was abolished in the *si*FoxC1 IHs (Figure 3(a)). The quantitative analysis of the inflammatory cells showed that the inflammatory response in the *si*FoxC1 IHs was greater compared to that of the CON IHs (Figure 3(c)). Both of the *ad*FoxC1 IHs significantly reduced the inflammatory response compared to the other IHs; cotreatment of MSC transplantation and *ad*FoxcC1 transfection had the best inflammation-inhibiting effects (Figure 3(c)), accompanying the increase of viable cardiomyocytes. However, *si*FoxC1 abolished these ameliorations (Figure 3(d)). The quantitative indices for inflammation, i.e., myocardium MPO and ROS, were highest in the *si*FoxC1 IHs receiving PBS injection and were the lowest in the *ad*FoxC1 IHs receiving MSC therapy (Figures 3(e) and 3(f)). Quantitative analysis of TTC staining showed that, compared with PBS injection alone, *ad*FoxC1 transfection or MSC therapy reduced infarct size significantly, and the effect was greatest in the MSC-treated *ad*FoxC1 IHs. However, *si*FoxC1 diminished this effect (Figure 3(g)). The collagen-rich myocardial scar in the infarcted wall was stained blue, whereas the viable myocardium was stained red. Light microscopy revealed more loss of crossstriations with the appearance of focal inflammation and bleeding and interstitial tissue edema in the rats receiving PBS therapy compared with those receiving MSC therapy, which was deteriorated in the *si*FoxC1 IHs but was alleviated in the *ad*FoxC1 IHs. Moreover, more salvaged myocardial tissue and less collagen were observed in the MSC-treated *ad*FoxC1 IHs (Figure 3(b)). Statistical analysis showed that the *ad*FoxC1 plus MSC-treated IHs had the greatest decrease of collagen content compared with other IHs. However, *si*FoxC1 cancelled this decrease (Figure 3(h)). Hence, these results indicate that FoxC1 may form an anti-inflammatory and antifibrosis niche that contributes to improving MSC function.

**Figure 4:**
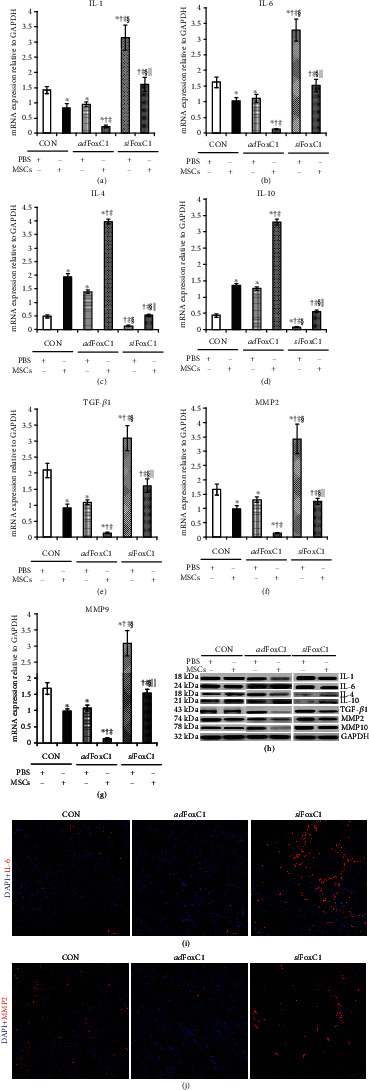
FoxC1 overexpression reduces inflammatory/fibrotic factor expression in the niche of the engrafted MSCs. qPCR analysis of the mRNA expression of the inflammatory factors IL-1 (a) and IL-6 (b), anti-inflammatory factors IL-4 (c) and IL-10 (d), and fibrotic factors TGF-*β*1 (e), MMP2 (f), and MMP9 (g) in the CON IHs, the *ad*FoxC1 IHs, or the *si*FoxC1 IHs treated with PBS or MSCs for 30 days. (h) Heart tissue lysates were immunoprecipitated with antibodies against these factors. Immunoprecipitates were analyzed by immunoblotting with antibodies against IL-1, IL-6, IL-4, IL-10, TGF-*β*1, MMP2, and MMP9. GAPDH served as the loading control. MSC transplantation resulted in a significant decrease in the expression levels of these factors, and this reduction was more pronounced in the MSC-treated *ad*FoxC1 IHs. *si*FoxC1 intervention canceled it. (i, j) Immunofluorescence images depicting loss of representative inflammatory factor IL-6 (i) and fibrotic factor MMP2 (j) after transplantation of MSCs into the *ad*FoxC1 IHs. The nuclei were stained with DAPI and revealed as blue; the cytoplasm was stained as red with anti-IL-6 or MMP2. Scale bars = 50 *μ*m. All graphical data are the mean ± SEM. ^∗^*p* < 0.05 vs. PBS in the CON IHs, ^†^*p* < 0.05 vs. MSCs in the CON IHs, ^‡^*p* < 0.05 vs. PBS in the *ad*Foxc1 group, ^§^*p* < 0.05 vs. MSCs in the *ad*Foxc1 IHs, and ^||^*p* < 0.05 vs. PBS in the *si*Foxc1 IHs (CON IHs: PBS, *n* = 6, and MSCs, *n* = 7; *ad*Foxc1: PBS, *n* = 10, and MSC*s*, *n* = 13; *si*FoxC1: PBS *n* = 4, and MSCs, *n* = 6); Student's *t*-test.

Real-time qPCR and immunoblotting were used to examine the expression levels of the inflammatory factors IL-1 and IL-6, the anti-inflammatory factors IL-4 and IL-10, and the fibrosis-related factors TGF-*β*1, MMP2, and MMP9. FoxC1 transfection alone or MSC transplantation alone decreased the mRNA and protein expression levels of IL-1, IL-6, TGF-*β*1, MMP2, and MMP9 to some extent, and the reduction was greatest in the MSC-treated *ad*FoxC1 IHs. By contrast, FoxC1 overexpression dramatically enhanced both mRNA and protein expression levels of IL-4 and IL-10. However, *si*FoxC1 upregulated these factors: IL-1, IL-6, TGF-*β*1, MMP2, and MMP9, and downregulated IL-4 and IL-10. No significant difference was seen between the PBS-treated CON IHs and the MSC-treated *si*FoxC1 IHs, and the MSC-treated CON IHs and the PBS-treated *ad*FoxC1 IHs (Figures 4(a)–4(h)). Immunofluorescence confirmed the FoxC1-mediated inhibition of the inflammation-related target gene IL-6 and fibrosis-related gene MMP2 in the different niches of engrafted MSCs (Figures 4(i) and 4(j)). These experiments demonstrate mechanistically that FoxC1 overexpression mediates activation of anti-inflammatory and antifibrotic signaling.

### 3.4. Sustained Upregulation of FoxC1-Mediated Proangiogenic Factors Was Attributed to Host Vascular Niche

**Figure 5:**
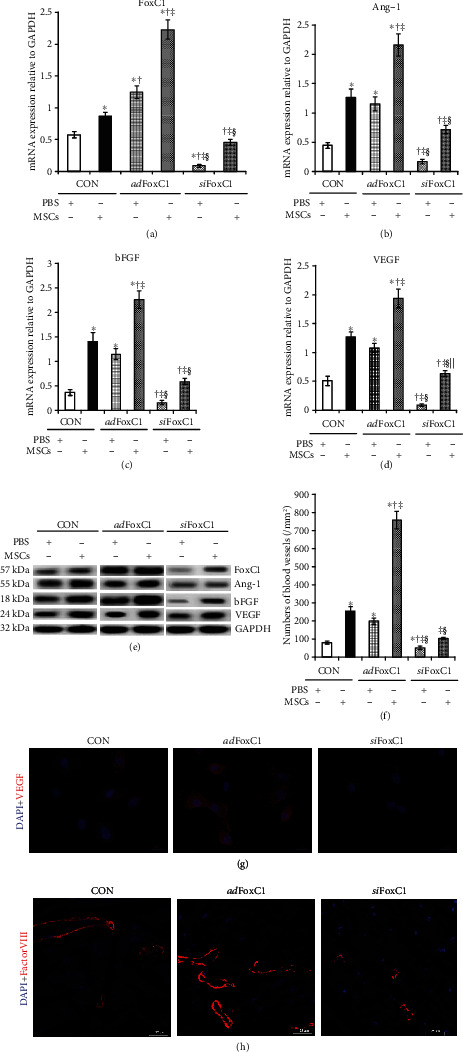
FoxC1 contributes to MSC therapy-induced angiogenesis in the *ad*FoxC1 IHs. (a) FoxC1 mRNA expression in PBS or MSC-treated IHs in the presence/absence of FoxC1 transfection/knockdown. (b–d) mRNA expression of Ang-1 (b), bFGF (c), and VEGF (d) in the CON IHs, the *ad*FoxC1 IHs, or the *si*FoxC1 IHs after cell therapy. (e) FoxC1, Ang-1, bFGF, and VEGF protein expression in the IHs treated with PBS or MSCs in the presence/absence of *ad*FoxC1/*si*FoxC1. In the IHs, MSC therapy upregulated FoxC1, Ang-1, bFGF, and VEGF expression compared with PBS injection; FoxC1 pretransfection improved their expression, whereas *si*FoxC1 abolished it. (f) FoxC1 improvement of angiogenesis induced by MSC therapy in the *ad*FoxC1 IHs was determined by the number of factor VIII positive-staining vessels per mm^2^ under high-power field view. (g, h) Representative images of VEGF (g, red, scale bar = 20 *μ*m) or factor VIII (h, red, scale bar = 25 *μ*m) staining of cardiomyocytes and vessels in the infarct and peri-infarct areas from the MSC-treated IHs in the presence/absence of *ad*FoxC1/*si*FoxC1. The nuclei were stained with DAPI and revealed as blue. VEGF was mainly expressed by the blood vessels and cardiomyocytes in the MSC-treated IHs, especially in the animals that had received MSCs combined with FoxC1 transfection. Similarly, the greatest vascular density can be seen in the MSC-treated *ad*FoxC1 IHs. All graphical data are the mean ± SEM. ^∗^*p* < 0.05 vs. PBS in the CON IHs, ^†^*p* < 0.05 vs. MSCs in the CON IHs, ^‡^*p* < 0.05 vs. PBS in the*ad*Foxc1 group, ^§^*p* < 0.05 vs. MSCs in the *ad*Foxc1 IHs, and ^||^*p* < 0.05 vs. PBS in the *si*Foxc1 IHs (CON IHs: PBS, *n* = 6, and MSCs, *n* = 7; *ad*Foxc1: PBS, *n* = 10, and MSC*s*, *n* = 13; *si*FoxC1: PBS *n* = 4, and MSCs, *n* = 6); Student's *t*-test.

To determine the levels of expression of FoxC1-related proangiogenic factors in the IHs after cell therapy, we measured the mRNA and protein levels of its transcription reporter FoxC1 and its target factors Ang-1, bFGF, and VEGF in the myocardium using qRT-PCR, western blotting, and immunofluorescence. At day 30, MSC therapy had induced an overall increase in their expression; FoxC1 upregulation magnified this effect: in comparison with cell therapy alone or Foxc1 transfection alone, MSC therapy in the IHs pretransfected with *ad*FoxC1 induced the greatest increase in FoxC1, Ang-1, bFGF, and VEGF mRNA and protein expressions. *si*FoxC1 significantly attenuated this increase (Figures 5(a)–5(e)). Immunofluorescence showed that proangiogenic cytokines, e.g., VEGF, were detected mainly in the vascular ECs and ischemic lesions in the IHs pretreated with *ad*FoxC1, but their expression levels were significantly decreased in the *si*FoxC1 IHs (Figure 5(g)). Tissue sections were stained for anti-factor VIII antibody to detect blood vessel density. MSC-treated IHs had more blood vessels than the PBS-treated IHs, and the MSC-treated *ad*FoxC1 IHs had the greatest blood vessel density with 2.0-fold and 2.8-fold differences compared with the CON IHs receiving MSCs alone and the PBS-treated *ad*FoxC1 IHs, respectively. *si*FoxC1 appeared to prevent this increase in vessel density (Figures 5(f) and 5(h)). Collectively, these findings suggest that FoxC1 induces the initial upregulation of proangiogenic cytokine expression and that the cooperation between FoxC1 and MSCs appears to be more beneficial to angiogenesis in the ischemic niches.

### 3.5. FoxC1 Overexpression Ensures MSC Anchoring to Prevent Its Loss during Ischemia

**Figure 6:**
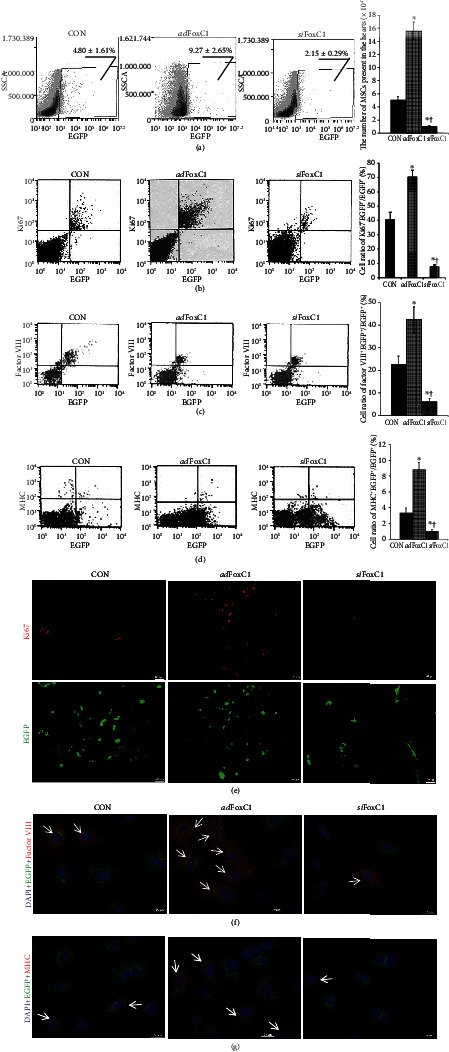
FoxC1 is required for survival and neovascularization of transplanted MSCs. MSCs were infected with a reporter retrovirus expressing EGFP before transplantation and were recognized as EGFP-positive cells. (a–c) FACS assessment of the (a) percentages of EGFP-positive cells (EGFP^+^) relative to the whole ventricular cell population and the number of EGFP^+^MSCs present in the IHs after 30 days of transplantation; (b) percentages of Ki67 (proliferation index marker) and EGFP double-positive cells (Ki67^+^EGFP^+^) relative to the whole EGFP^+^ population, factor VIII and EGFP double-positive cells (factor VIII^+^EGFP^+^) relative to the whole EGFP^+^ population (c), or myosin heavy chain (MHC) and EGFP double-positive cells (MHC^+^EGFP^+^) relative to the whole EGFP^+^ population (d) as assessed by FACS. Three images in the left row represent the representative phenotypes of gated (a) EGFP^+^ in total cardiomyocytes, (b) Ki67^+^EGFP^+^, (c) MHC^+^EGFP^+^ cells, and (d) factor VIII^+^EGFP^+^ cells evaluated by FACS in transplanted MSCs. The image in the far right row represents the statistical results of FACS. All graphical data are the mean ± SEM. ^∗^*p* < 0.05 vs. MSC-treated CON IHs, ^†^*p* < 0.05 vs. MSC-treated *ad*FoxC1 IHs (*n* = 5 per group). (e–g) Immunofluorescence staining showing that transplanted cells expressed Ki67 (e), factor VIII (f), and MHC (g). The transplanted cells were prelabeled with EGFP (green); the nuclei were stained with DAPI (blue). Ki67 (red) was mainly expressed in the nuclei and the nuclear membrane. Scale bars = 100 *μ*m. The cytoplasm was stained with anti-factor VIII antibody (red). The myocardium was stained with anti-MHC antibody (red). Scale bars = 50 *μ*m. Engrafted EGFP-prelabeled cells expressing Ki67, factor VIII, or MHC were the most numerous in the *ad*FoxC1 IHs and were lowest in *si*FoxC1-transfected IHs (arrows).

We investigated the relationship between FoxC1 signaling and transplanted MSC survival/engraftment. The detection of EGFP-labeled MSCs was confirmed by FACS and immunofluorescence. Cell retention 30 days after the transplantation of MSCs into the CON IHs, *ad*FoxC1 IHs, or *si*FoxC1 IHs is shown in Fig. S4. EGFP^+^ cells were found more in the *ad*FoxC1 IHs than in the CON IHs, whereas very few EGFP ^+^ cells were found in the *si*FoxC1 IHs (Fig. S4a). The number of EGFP^+^MSCs was much higher in the *ad*FoxC1 IHs compared with CON IHs or *si*FoxC1 IHs (Fig. S4b). Consistently, FACS demonstrated that the retention of survival MSCs reduced significantly at 30 d after the injection of 5 × 10^6^ MSCs; 1.01% of the initially engrafted cells were detected in the CON IHs and further declined to ~0.2% in the *si*FoxC1 IHs. However, FoxC1 transfection significantly increased the EGFP^+^MSCs in the *ad*FoxC1 IHs by 3.12% of the initially engrafted cells (Figure 6(a)), indicating an increase in cell engraftment in the overexpression of FoxC1.

Furthermore, more cells which coexpressed EGFP and the proliferation marker Ki67 were seen in the *ad*FoxC1 IHs than in the CON IHs or in *si*FoxC1 IHs (Figures 6(b) and 6(e)). Taken together, these data suggest that FoxC1 signaling functions in part to ensure adequate engraftment of MSCs to prevent their loss during ischemia.

Genetically, EGFP-targeted in vivo cell tracking could help to clarify their reparative status in inflammation, fibrogenesis, and myocardial regeneration. Immunofluorescence imaging of transverse heart sections immunostained for myosin heavy chain (MHC) revealed that EGFP expression colocalized with MHC staining in the IHs (Fig. S4a), and the number of EGFP^+^MHC^+^ cells was much more in the *ad*FoxC1 group than in the CON group and the *si*FoxC1 group (Fig. S4c), suggesting that FoxC1 enhanced the incorporation of EGFP^+^MSCs into cardiomyocytes of the infarcted hearts. The effect of FoxC1 upregulation on the differentiation of the transplanted cells was also evaluated by FACS and immunofluorescence in EGFP-positive cells isolated from the MSC-treated IHs. There was a 1.9-fold increase in the expression of the vascular endothelial cell marker factor VIII in the MSC-treated *ad*FoxC1 IHs compared with the MSC-treated CON IHs, but the expression of MHC was much lower in both groups, and *si*FoxC1 appeared to decrease these protein expressions significantly (Figures 6(c), 6(d), 6(f), and 6(g)). Therefore, FoxC1 is mainly required to ameliorate vessel differentiation of MSCs in the ischemic niches.

### 3.6. FoxC1 Reduces Macrophage and Myofibroblastic Differentiation of MSCs

**Figure 7:**
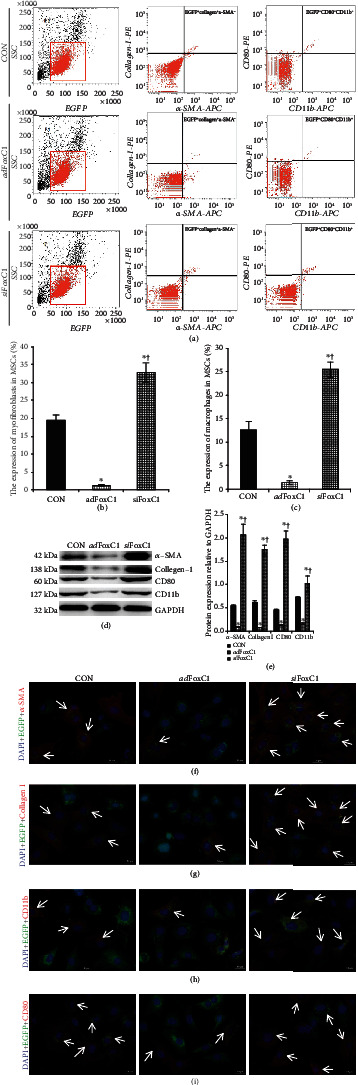
Overexpression of FoxC1 reduced MSC differentiation into myofibroblasts and macrophages in the IHs. (a–c) The expression of macrophage and myofibroblast markers in the EGFP-positive (EGFP^+^) cells was examined by FACS. (a) Three images represented representative phenotypes of gated EGFP^+^ in total cardiomyocytes, *α*-SMA^+^collagen I^+^, or CD80/CD11b cells evaluated by FACS in transplanted MSCs. (b, c) The graphical images represent the statistical results of FACS. (d) Equal protein loading was assessed by immunoblotting for myofibroblast marker proteins, including *α*-SMA, collagen I, and macrophage marker proteins, CD80 and CD11b, in transplanted MSCs. (e) Quantitative analysis of *α*-SMA, collagen I, CD80, and CD11b protein expression in IHs treated with MSCs in the presence or absence of the *ad*FoxC1 or *si*FoxC1. All graphical data are the mean ± SEM. ^∗^*p* < 0.05 vs. the MSCs engrafted into the CON IHs and ^†^*p* < 0.05 vs. the MSCs engrafted into the *ad*FoxC1 IHs (*n* = 5 per group). (f–i) Representative images of immunofluorescence staining for *α*-SMA (f, red), collagen I (g, red), CD80 (h, red), or CD11b (i, red) in EGFP- (green) prelabeled MSCs engrafted as mentioned previously. Nuclei were stained with DAPI (blue). Scale bar : 50 mm. Note that FoxC1 overexpression strongly reduced the expression of myofibroblast markers and macrophage markers in the *ad*FoxC1 group, whereas significant increases in the levels of these proteins were observed in the *si*FoxC1 group (arrows).

In addition to angiogenesis and myocardiogenesis, MSCs also have myofibroblastic differentiation and inflammation properties, dependent on extracellular niches [14–16]. To test the transformation of MSCs into myofibroblast and inflammatory cells in the ischemic niches, we examined EGFP-labeled MSCs in combination with specific markers for the myofibroblast and macrophage, *α*-SMA/collagen I and CD80/CD11b, using FACS, western blot, and immunocytofluorescence. FACS demonstrated that the frequency of *α*-SMA^+^collagen I^+^ myofibroblasts and CD80^+^CD11b^+^ macrophages in the EGFP^+^ cell population was 13.6-fold or 7.0-fold lower, respectively, in the MSC-treated *ad*FoxC1 IHs compared with CON, but *si*FoxC1 significantly increased the frequency (Figures 7(a)–7(c)). Western blotting revealed lower *α*-SMA, collagen I, CD80, and CD11b expression in the *ad*FoxC1 as compared with CON, whereas the expressions of these marker proteins were significantly higher in the MSCs from the *si*FoxC1 IHs (Figures 7(d) and 7(e)). Immunofluorescence showed that compared with CON, FoxC1 overexpression significantly reduced expressions of *α*-SMA, collagen I, CD80, and CD11b in MSCs from the *ad*FoxC1 IHs. However, depletion of FoxC1 canceled this reduction in transformation, and much higher expressions of these markers were seen in the *si*FoxC1 group (the *si*FoxC1+MSCs group) (Figures 7(f)–7(i)). The results showed that FoxC1 contributes to inhibiting transformation of myofibroblast and inflammation of MSCs in the IHs.

## 4. Discussion

MSCs can significantly improve tissue repair by conferring regeneration potential in injured cardiovascular tissue; however, maintaining sufficient functional MSCs in the IHs for autologous stem cell therapy is challenging. While various measures for improving stem cell viability and function have been extensively investigated, little attention has been paid to the effects ischemia exerts on the stem cell niche. Here, we identify FoxC1 as an endothelial transcriptional effector that promotes survival and vascular stability of MSCs in the ischemic niche. FoxC1 controls the expression of the ischemia-mediated proangiogenic cytokines and inflammatory/fibrotic factors in the vascular niche, promoting angiogenesis, inhibiting inflammation and fibrosis, impeding the development of HF, preserving LV function and dimensions, and preventing infarct expansion induced by MSC therapy.

Self-renewal, activation, and differentiation of stem cells could be regulated by the cells and proteins that constitute the extracellular environment (or “niche”) [17]. Common components of stem cell niches are composed of neighboring cells, their differentiating daughters, and growth factors. We first observed that proangiogenic cytokines, including Ang-1, bFGF, and VEGF, were more abundantly expressed in the IHs transfected with FoxC1 15 d after MI. Consistent with the changes in these cytokines, the blood vessel density was greater in the *ad*FoxC1 IHs compared with the hearts without FoxC1 transfection, and *si*FoxC1 reduced the blood vessel density of the IHs. These data suggested that FoxC1 might provide a vascular niche, which might enhance angiogenesis of the IHs.

The transcription factor FoxC1 has been identified as one of the few genes with dynamic histone modification patterns in hair follicle stem cells [18], but its expression and function in MSCs are unknown. Here, transplantation of MSCs into FoxC1-transfected vascular niches resulted in greater improvement in the survival rate and cardiac remodeling of MI rats via decreasing myocardial inflammatory and fibrosis and increasing angiogenesis in comparison with MSC therapy alone. In addition, because we found, in the IHs without cell injection (PBS injection), that the left ventricular pathological structure was improved to some extent in those with FoxC1 transfection alone (*ad*FoxC1) than in others without FoxC1 transfection (CON), it is possible that the predevelopment of angiogenesis after FoxC1 transfection is related to the improvement of myocardial viability. The importance of the vascular niche in preclinical cases of stem cell therapy has also been shown in our previous articles where the beneficial effects of CD34^+^ progenitor cells or endothelial progenitor cells (EPCs) after MI on cardiac repair were magnified in the swine with autologous preexisting angiogenesis [4, 9]. All these data suggested that FoxC1-induced vascular niches might magnify the beneficial effects on cardiac repair induced by cell therapy after MI.

Although FoxC1-dependent mesenchymal signaling drives progenitor cell proliferation and growth in early embryonic brain development [19], the mechanism that reinforces MSC survival and function in the ischemic niche is unclear. In this present study, we first show that FoxC1 transfection into IHs improved MSC survival and proliferation, which is associated with the increase of proangiogenic and anti-inflammatory cytokines and decrease of inflammatory and fibrotic factors, consisting of data from the recent study by Lee et al. [20] identifying that activated FoxC1 promoted cell proliferation and self-renewal of multipotent arachnoid-pia mater stem cells. This improvement effect of FoxC1 has conversely been confirmed by depletion of FoxC1 inducing decreased viability and function of MSCs. In this study, knockdown of FoxC1 caused significant changes in these factors, including inhibiting expression of proangiogenic factors Ang-1, bFGF, and VEGF and anti-inflammatory factors IL-4 and IL-10 and increasing the expression of inflammatory and fibrotic factors, which caused significant worsening of inflammation and fibrosis, resulting in decreased proliferation, angiogenesis, and neovascularization. This indicates that FoxC1-induced loss of balance between inflammation, fibrosis, and angiogenesis appears to be defective maintenance of the vascular niche. Together, these findings imply that vascular niches with higher levels of FoxC1-mediated angiogenic cytokine expression and angiogenesis before cell therapy are superior microenvironments for MSCs engrafted into IHs and that FoxC1 plays a key role in maintaining these niches.

FoxC1 have also been found to regulate vessel specification and differentiation [21]. Although the MSCs are shown to differentiate into cells with myocardial and endothelial features, doubts exist regarding their unwanted differentiation [22]. This suggestion is confirmed by our present study. This is the first study to demonstrate that MSCs engrafted into the IHs overexpressed by *ad*FoxC1 transfection mainly exhibited vessel vascular differentiation but less myocardial differentiation and rare differentiation into inflammatory cells or fibroblasts. However, when engrafted into the *si*FoxC1 IHs, MSCs showed higher transformation of macrophages and fibroblasts, resulting in strong fibrosis and inflammation. Thus, the vascular formation of MSCs in the IHs appears to be inextricably linked with the extent of FoxC1expression. According to the dynamic changes of inflammation after MI, typing of MSC-transformed macrophages remains a future investigation, which may help us to better explain the relief of inflammation.

Recent findings demonstrate that a population of MSCs, termed CXC chemokine ligand (CXCL) 12-abundant reticular (CAR) cells or leptin receptor-expressing (LepR^+^) cells, are the major cellular components of niches for the hematopoietic stem cells (HSCs) and lymphoid progenitors, which express specific transcription factors and cytokines, essential for their niche functions [23]. In our present study, compared with the PBS-treated IHs, FoxC1 expression is upregulated in the MSC-treated IHs, which is associated with the simultaneous increase of proangiogenic factors that contribute to the positive regulation of cell survival of MSCs. However, this positive regulation was interrupted by the depletion of FoxC1. In the *si*FoxC1 IHs, low expression of FoxC1 is associated with low survival of MSCs. All these results indicate positive reciprocal feedback in the ischemic niche among FoxC1 and MSCs, where FoxC1 is required for maintaining the positive FoxC1-MSC loop (Supplementary Fig. S5).

In summary, our findings best fit a model whereby FoxC1 induces vascular niches after MI. This in turn increases MSC sensitivity to ischemic injury by upregulating the proangiogenic and anti-inflammatory cytokines and decreasing inflammatory and fibrotic factor expression, further accelerating myocardial repair. In the ischemic environment, prudence in conserving FoxC1 expression appears to be essential for maintaining MSC numbers and preserving their vessel formation during MI. In this manner, the niche that is enriched with FoxC1 and angiogenesis may serve as an optimal host microenvironment for transplanted stem cells post-MI, and investigation of such adaptive mechanisms should provide a potential therapeutic target for the future treatment of ischemic diseases.

## Data Availability

1. The data used to support the findings of this study are included within the article and the supplementary information file(s). 2. The data used to support the findings of this study are also available from the corresponding author upon request.

## Supplementary Materials

Supplementary Materials The supplementary file includes research design and methods. Table S1 and Table S2: list the qRT-PCR primers and the antibodies. Fig. S1, Fig. S2, Fig. S3, Fig. S4, and Fig. S5: present the treatment flowchart, the expression time curve of FoxC1-related vascular growth factors, the MSC surface marker expression, local engraftments and myocardiogenesis of MSCs, and the model of FoxC1 mediation of myocardial repair of engrafted MSCs, respectively.
