# Supplementary material for: FoxC1-Induced Vascular Niche Improves Survival and Myocardial Repair of Mesenchymal Stem Cells in Infarcted Hearts
Source: Oxid Med Cell Longev. 2020 Jul 4;2020:7865395. doi: 10.1155/2020/7865395 (PMC7490631; doi:10.1155/2020/7865395)
Supplement: Supplementary Materials — The supplementary file includes research design and methods. Table S1 and Table S2: list the qRT-PCR primers and the antibodies. Fig. S1, Fig. S2, Fig. S3, Fig. S4, and Fig. S5: present the treatment flowchart, the expression time curve of FoxC1-related vascular growth factors, the MSC surface marker expression, local engraftments and myocardiogenesis of MSCs, and the model of FoxC1 mediation of myocardial repair of engrafted MSCs, respectively. [file 7865395.f1.pdf]

Research design and methods

Antibodies and other reagents

Transwell six-well plates were from COSROR. The ABsolute QPCR SYBR Green premix was from Takara. Supplementary **Tab. S1** presents the sequences of primers used in this study. Supplementary **Tab. S2** lists the details of the antibodies.

Animals

Inbred Lewis rats (18 months old) were used. The Animal Care and Use Committee of Yangpu Hospital, Tongji University School of Medicine, approved all animal experiments, which were in compliance with the Guide for the Care and Use of Laboratory Animals published by the National Academy Press.

FoxC1 transfection and siRNA knockdown

Adenoviral vector encoding the recombinant bovine FoxC1 gene under the control of a cytomegalovirus promoter (*ad*FoxC1) and Ad.Null (CON) were used, as described previously[1]. To knock down FoxC1 expression, transfection was performed with siRNA targeted to FoxC1 as previously described[2]. In brief, to formulate the lipid-siRNA complex, siRNA (Origene Technologies) and Lipofectamine 2000 (Invitrogen) were diluted separately in Opti-MEM (Invitrogen).

MI model and FoxC1 transfection

Infarcted hearts (IHs) were induced in inbred Lewis rats, obtained from the Shanghai Animal Administration Center, by ligating the left anterior descending (LAD) coronary artery. Animals with an ejection fraction (EF)<70% and fractional shortening (FS)<35% evaluated by echocardiography after induction of IHs were

selected. To investigate the effects of FoxC1 on the ischemic niche, the animals were randomly divided into three groups corresponding to Foxc1 transfection status: knockdown of FoxC1 by transfecting the IHs with vectors encoding FoxC1 siRNA (*si*FoxC1 group), no-intervention, and overexpression of FoxC1 by transfecting the IHs with vectors encoding FoxC1 (*ad*FoxC1 group). No-intervention rats were transfected with control vectors (CON). After 15 days post-induction, twenty animals in each group were sacrificed to exactly valuate the FoxC1-induced vascular environment at the tissue, cellular, and molecular levels. Meanwhile, other animals received either vehicle (phosphate-buffered saline [PBS]) or MSCs injection.

Analysis of the FoxC1-mediated niches

The base myocardial tissues were used for qRT-PCR, western blot, and immunohistochemistry evaluation of the expression of FoxC1 and proangiogenic cytokines (Ang-1, bFGF, VEGF). To quantify blood vessel density, additional slides were prepared from the animals in each subgroup. The sections were stained with anti–factor VIII antibody and counterstained with an immunoperoxidase kit (Vector Labs). Ten fields per slide were randomly chosen from infarct portions that were bordered by non-infarct portions along the LAD. Blood vessel density was expressed as the number of factor VIII^{+} ECs per square millimeter[3]. The vessels were defined as round or elliptical structures with a central lumen lined by cells staining positively for factor VIII. A pathologist blinded to the group identities evaluated the capillary density by counting vessels in the selected areas.

Enzyme-linked immunosorbent assays (ELISA)

The levels of FoxC1, Ang-1, bFGF, and VEGF in supernatant of heart tissues were measured by ELISA using a duoset methodology (R&D Systems; Minneapolis, MN). Briefly, after standard procedures, the tissue-free samples were pipetted into the wells of the microtitre plates, specific horseradish peroxidase-linked polyclonal antibodies were added and immunoreactive levels of FoxC1, Ang-1, bFGF, and VEGF were determined. Values below the detection limit were assumed as zero.

Real-time RT-PCR

Total RNA was isolated from homogenized heart tissue or cultured cells using TRIzol (Gibco BRL). The RNA was reverse-transcribed with a TaqMan cDNA Synthesis Kit (Applied Biosystems) and amplified on a TaqMan 7500 (Applied Biosystems). Supplementary **Tab. S1** lists the primer and probe sequences. Expression was calculated via the comparative threshold cycle (Ct) method and normalized to that of GDPDH, which was included as an internal control; thresholds for the individual reactions were determined using ABI Prism SDS 2.0 data processing software (Applied Biosystems).

Western blotting

To confirm the protein expression of the genes of interest, western blotting was performed as described previously[4] with modifications. Briefly, protein extracts (100 μg per sample) were separated using sodium dodecyl sulfate–polyacrylamide gel electrophoresis (SDS-PAGE; Bio-Rad Laboratories) and electrotransferred onto polyvinylidene difluoride (PVDF) membranes (GE Healthcare). Samples were probed with antibodies (Supplementary **Tab. S2**) against FoxC1, Ang-1, bFGF, VEGF, IL-1,

IL-6, IL-4, IL-10, factor VIII, TGF-β1, MMP2, MMP9, Collagen I, α-SMA, CD80, and CD11b. GAPDH served as a positive control. The target protein levels were determined as the ratios of the target protein/GAPDH using ImageQuant software (GE Healthcare).

Isolation, expansion, purification, and FACS of MSCs

MSCs were isolated and purified from isolated MNCs, and cultured via the adherent culture method as our previously described[5, 6]. Briefly, MNCs were cloned via limited dilution, and expanded in MSC complete medium (Iscove's modified Dulbecco medium: IMDM with 20% fetal bovine serum, 2 mM L-glutamine, penicillin [100 U/ml] and streptomycin [100 μg/ml]). At 80% confluence, cells were collected, and their purity and character were determined using FACS. The characters of the MSCs were confirmed with antibodies against CD34, CD44, CD71, CD90, CD147, SH2, SH3, CD45, and CD133. Mouse IgG1, IgG2a, and IgG2b (Becton Dickinson) were used as isotype controls, and marker expression was evaluated using a FACS apparatus as mentioned above. Cell viability was evaluated via the propidium iodide exclusion assay and by flow cytometry. FACS showed that 86.5%-99.4% of MSCs expressed CD44, CD71, CD90, CD147, SH2, and SH3, whereas they expressed less than 5% of CD34, CD45, and CD133 (**Fig. S3**).

EGFP labelling

Before transplantation, MSCs were transfected with a lentiviral vector containing enhanced GFP (EGFP) cDNA, as described previously[7]. More than 70% of MSCs were EGFP-positive, as determined by flow cytometry.

Cell transplantation and animal grouping

15d after establishing the FoxC1-induced vascular niche models, the animals were randomized to receive PBS injection or MSCs therapy. The total volume of PBS or cell suspension injected was ~100 μL, and each animal in the cell treatment groups received a total of 5 × 10^6 cells. Injections were administered to the infarct and peri-infarct regions at four sites separated by 1–2 cm. Then, all animals were randomly divided into six groups (n = 20 per group) (Supplementary **Fig. S**1): CON rats: PBS injection in the CON IHs [(+) PBS], MSCs transplantation into the CON IHs [(+) MSCs]; *ad*FoxC1 rats: PBS injection in the *ad*FoxC1 IHs [(+) PBS], MSCs transplantation into the *ad*FoxC1 IHs [(+) MSCs]; *si*FoxC1 rats: PBS injection in the *si*FoxC1 IHs [(+) PBS], MSCs transplantation into the *si*FoxC1 IHs [(+) MSCs]. To minimize postoperative pain, 2.5% bupivacaine was sprayed at the incision point immediately before closure, and buprenorphine hydrochloride (0.03 mg/kg) was administered intramuscularly. After the final layer of skin was closed, triple antibiotic ointment (neomycin sulfate, polymyxin B sulfate, bacitracin zinc) was applied to the surgical wound.

Follow-up

All rats underwent echocardiography to evaluate cardiac functions before transplantation and at 30 days post-cell therapy, and were closely observed up to death to evaluate their survival curves.

Echocardiography

Under general anesthesia as described above, rats underwent echocardiography in a

7.5-MHz phased-array transducer (Acuson Sequoia 256) operated by an experienced technician blinded to treatment group identity; two-dimensional images were obtained at the mid-papillary and apical levels. Left ventricular end-diastolic volume, internal diameter, anterior wall thickness, and posterior wall thickness at diastolic phase (LVEDV, LVEDD, LVAWd, and LVPWd, respectively) were measured using the biplane area-length method. LV fractional shortening (FS) was calculated according to the modified Simpson method: FS (%) = [(LVIDd-LVIDs)/LVIDd] × 100, where LVID is LV internal dimension, s is systole, and d is diastole. All measurements were averaged for three consecutive cardiac cycles.

MPO and ROS detection

At the end of each study, the heart was removed and divided into three transverse slices from the base, mid-region, and apex. The apical slices were homogenized in saline (per 100 mg tissue in 0.9 ml saline). After 15-min centrifugation at 3000 ×*g*, the supernatant was collected and stored at -70℃ until used. Myocardium myeloperoxidase (MPO) and reaction oxygen species (ROS) were detected according to the manufacturer's instructions (A044, A018, NJBI). The peroxide-sensitive fluorescent probe 2′,7′-dichlorodihydrofluorescein diacetate (Molecular Probes) was used to assess the generation of intracellular ROS as described previously[8]. This compound is converted by intracellular esterases to 2′,7′-dichlorodihydrofluorescein (DCF), which is then oxidized by hydrogen peroxide to highly fluorescent DCF. Differential interference contrast images were obtained simultaneously using an Olympus inverted microscope with ×40 Aplanfluo objective and an Olympus

fluoview confocal laser-scanning attachment. The DCF fluorescence was measured with an excitation wave length of 488 nm of light, and its emission was detected using a 510–550-nm bandpass filter. The change in MPO absorbance was measured at 460 nm using a spectrophotometer. One unit of MPO was defined as the quantity of enzyme required to hydrolyze peroxide at a rate of 1 mmol/min at 25 ℃.

Assessment of inflammation, myocardial infarct size, and collagen content

The mid-region areas in each group were used to evaluate the myocardial infarct size and collagen density by triphenyltetrazolium chloride (TTC) staining (Sigma) and Masson's trichrome stain as previously described[9]. The sections were stained at 37 ℃ for 15 min with 1.5% TTC in PBS and fixed with 4% paraformaldehyde solution overnight. The respective TTC-stained (red, normal) and non-stained (white, infarct) areas were measured digitally using Image-Pro Plus 6.0 software (Media Cybernetics). The percentage infarct area was used to estimate the extent of each myocardial infarct. In Masson's trichrome staining, the collagen-rich myocardial scar in the infarcted wall stains blue, whereas viable myocardium stains red. The collagen density was expressed as the proportion of collagen deposition or scar tissue to normal LV myocardium. H&E staining was performed on each LV cross section to examine myocardial injury and inflammatory responses. Inflammation cells and viable myocardium were quantified from 4 separate fields/animal using Image-Pro Plus analysis software (version 6.2, MediaCybernetics, Bethesda, MD) and expressed as the percent area stained.

Immunohistochemistry, immunohistofluorescence, and immunocytofluorescence

The peri-infarct region was paraffin-embedded and cut into serial 10-μm slices. The sections were stained with antibodies against the following (Supplementary **Tab. S2**): FoxC1, Ang-1, bFGF, VEGF, factor VIII, IL-6, and MMP2, and counterstained with an immunoperoxidase kit (Vector Labs) or 4,6-diamidino-2-phenylindole (DAPI; Roche Diagnostics, Basel, Switzerland). A pathologist blinded to treatment group identity counted the vessels in 10 randomly selected fields per section at 10 sections per heart. Vascularity was expressed as the number of factor VIII$^+$ vessels per square millimeter[6].

Light microscopy images were obtained for each coverslip. For immunocytofluorescence staining prepare a cell culture dish plate with glass cover slips and coated with laminin solution (final concentration 50 μg/ml laminin in PBS). For immunofluorescence staining, a cell culture dish plate was prepared with glass cover slips and coated with laminin solution (final concentration 50 μg/ml laminin in PBS). The isolated myocytes or EGFP$^+$cells were plated by using a microscope. The cells were cultured adhere to cover slip for 12h at 37 ℃ in 2 % CO_2. Then, the solution was removed, and immunocytofluorescence staining was immediately started with a standard staining procedure protocol as previously described[10]. The cells were fixed with fresh 4% paraformaldehyde in PBS. Cells were incubated with primary antibodies at 1:100–500 dilution in 10% NGS (normal goat serum)/PBS overnight at 4℃. The primary antibodies used were against the following (Supplementary **Tab. S2**): Ki67, factor VIII, MHC, Collagen I, α-SMA, CD80, and CD11b. The secondary antibodies were species-appropriate immunoglobulins (Ig) linked to

tetramethylrhodamine isothiocyanate (TRITC) or fluorescein isothiocyanate (FITC) (Invitrogen). Vascular cell differentiation of transplanted MSCs into the IHs was evaluated by positive EGFP and factor VIII double staining using flow cytometry and immunocytofluorescence. All cells were then stained with 10 μg/ml DAPI (Santa Cruz Biotechnology) in PBS for 5 min. All immunocytofluorescence experiments included control cells on coverslips processed in parallel with the omission of a primary antibody; in these preparations, no signal was observed in the red or blue channel.

FACS of engraftment, proliferation, differentiation of transplanted MSCs

Cells were collected from the left ventricles of five randomly selected hearts per experimental group, as previously described[4]. Briefly, the heart was excised and perfused retrogradely with Ca^{2+}-free perfusion buffer. The left ventricle was minced in collagenase, and the solution was filtered through a nylon mesh. The collected cells were washed with PBS and analyzed with a flow cytometry apparatus (Becton Dickinson). Engraftment was evaluated by determining the proportion of cells that expressed EGFP. Then, $EGFP^+$ cells were sorted by FACS. The cells (2×10^5/ml) were then incubated with 4 ml antibodies against Ki67, factor VIII, or MHC, α-SMA, collagen I, CD80, or CD11b. Proliferation was evaluated by calculating the proportion of cells that expressed both EGFP and Ki67; vascular, cardiomyocyte, myofibroblast, or macrophage differentiation was evaluated by calculating the proportion of cells that expressed both EGFP and factor VIII, MHC, α-SMA /collagen I, or CD80/CD11b.

Statistical analysis

Results are expressed as mean averages ± SEM. The surgical team and investigators were blinded to the treatment and results until the study analysis was completed, results tabulated, and the reports were released. Statistical analysis was performed using IBM SPSS 20.0 (IBM SPSS, Inc.). For quantitative variables, the means and standard deviations were calculated. Discrete variables are presented as percentages. Comparisons were performed with the χ^2 or Fisher's exact test for discrete variables and by the unpaired *t*-test, Wilcoxon sign-rank test, or analysis of variance for continuous variables. When comparing continuous variables between groups, we used analysis of variance followed by Student's *t*-tests, when appropriate. Chi-square analysis was used to compare animal survival rates between groups. A 95% confidence interval (CI) ($p < 0.05$) was considered significant.

References

1. Hirooka Y, Sakai K, Kishi T, Ito K, Shimokawa H, Takeshita A. Enhanced depressor response to endothelial nitric oxide synthase gene transfer into the nucleus tractus solitarii of spontaneously hypertensive rats. Hypertens Res 2003; 26(4): 325-331.
2. Tan X, Feng L, Huang X, Yang Y, Yang C, Gao Y. Histone deacetylase inhibitors promote eNOS expression in vascular smooth muscle cells and suppress hypoxia-induced cell growth. Cell Mol Med 2017; 21(9): 2022-2035.
3. Morishita T, Uzui H, Ikeda H, Amaya N, Kaseno K, Ishida K, Fukuoka Y, Lee JD, Tada H. Association of CD34/CD133/VEGFR2-Positive Cell Numbers with Eicosapentaenoic Acid and Postprandial Hyperglycemia in Patients with

Coronary Artery Disease. Int J Cardiol 2016; 221: 1039-1042.

4. Zhang S, Zhao L, Wang J,Chen N,Yan J, Pan X. HIF-2α and Oct4 HaveSynergisticEffects on Survival and Myocardial Repair of Very Small Embryonic–like Mesenchymal Stem Cells in Infarcted Hearts. Cell Death Dis 2017; 8(1): e2548.
5. Zhang S, Lu S, Ge J, Guo J, Chen P, Li T, Zhang P, Jia Z, Ma K, Liu Y, Zhou C, Li L. Increased heme oxygenase-1 expression in infarcted rat hearts following human bone marrow mesenchymal cell transplantation. Microvasc Res 2005; 69(1-2): 64-70.
6. Zhang S, Jia Z, Ge J, Gong L, Ma Y, Li T, Guo J, Chen P, Hu Q, Zhang P, Liu Y, Li Z, Ma K, Li L, Zhou C. Purified human bone marrow multipotent mesenchymal stem cells regenerate infarcted myocardium in experimental rats. Cell Transplant 2005; 14(10): 787-98.
7. Zhang S, Ge J, Zhao L, Qian J, Huang Z, Shen L, Sun A, Wang K, Zou Y. Host vascular niche contributes to myocardial repair induced by intracoronary bone marrow stem cells infusion in infarcted swine hearts. Stem Cells 2007; 25(5): 1195-1203.
8. Zhao L, Li S, Ge J, Sun A, Zou Y, Zhang S. Temporal changes in stem cell in the circulation and myocardium of mice with Coxsackie virus B3-induced myocarditis. Microvasc Res 2008; 75: 358-366.
9. Su F, Zhao L, Zhang S, Wang J, Chen N, Gong Q, Tang J, Wang H, Yao J, Wang Q, Zhong M, Yan J.Cardioprotection by PI3K-mediated signaling is required for

anti-arrhythmia and myocardial repair in response to ischemic preconditioning in infarcted pig hearts. Lab Invest 2015; 95(8): 860-871.

10. Köhncke C, Lisewski U, Schleußner L, Gaertner C, Reichert S, Roepke TK. Isolation and Kv channel recordings in murine atrial and ventricular cardiomyocytes. J Vis Exp 2013; (73): e50145.

Supplementary Table and Figure Legends

Supplementary Fig. S1. **Flow chart of animal MI preparation, gene transfection, cell transplantation, and grouping.**

Supplementary Fig. S2 ELISA assay of difference in FoxC1, Ang1, bFGF, and VEGF at days 1, 5, 10, 15, 20, 25, and 30 post-MI in the infarcted hearts (IHs) transfected with Foxc1vector (*ad*Foxc1), FoxC1 siRNAs (*si*FoxC1), or control siRNA duplexes (CON) immediately after MI induction. All data represent means±s.e.m. $p<0.05$: *vs the *ad*Foxc1-transfected IHs (*ad*Foxc1), †vs the CON IHs, ‡vs 1d, §vs 5d, $^{\|}$vs 10d, ¶vs 15d, $^{\#}$vs 20d, and $^{\$}$vs 25d post-MI in each group (n=20 in each group). Data are representative of at least 3 independent experiments.

Supplementary Fig. S3 Surface marker expression of rat MSCs. (**a**) Flow cytometry analysis of the immunophenotypic surface profiles for SH2, SH3, CD44, CD71, CD90, CD34, CD45, and CD133 of cultured MSCs. Histograms represent the counts of cells incubated with the relevant antibody. The logarithm on the x-axis represents the intensity of the fluorescent signal and the number of cells on the y axis. Third passage cultured MSCs were positive for the markers SH2, SH3, CD44, CD71, CD90, and CD147 but negative for CD34, CD45, and CD133. (**b**) Graph represents data based on three independent experiments.

Supplementary Fig. S4 Local engraftments and myocardiogenesis of EGFP labeled MSCs in the IHs. Representative fluorescence microscopy images of tissue sections showing the retention and myocardial differentiation of $EGFP^{+}$ MSCs at the injection site 30 days after transplantation (**a**). Myocardial differentiation (arrows) of

the EGFP-labeled (green) MSCs was confirmed with anti-MHC (myosin heavy chain) staining (red). Scale bar: 50 mm. Note that FoxC1 overexpression strongly increased the number of $EGFP^{+}$ MSCs and $EGFP^{+}MHC^{+}$ MSCs in the *ad*FoxC1 group, whereas significant decreases in the levels of these proteins were observed in the *si*FoxC1 group. Quantitative data showing the retention of $EGFP^{+}$ MSCs and $EGFP^{+}MHC^{+}$ MSCs s are shown in (**b** and **c**). Graph represents data combined from three independent experiments; results are presented as the mean+SEM. $^{*}p< 0.05$ vs. the CON group, $^{\dagger}p< 0.05$ vs. the *ad*Foxc1 group (CON, n = 7, *ad*Foxc1, n = 13; *si*FoxC1, n = 6), Student's t-test.

Supplementary Fig. S5 Positive reciprocal feedback in the ischemic niche among FoxC1 and MSCs. FoxC1 enhances proangiogenic cytokine expression and reduces the hypoxia-induced expression of inflammatory/fibrotic factors, leading to improved survival and angiogenesis of MSCs. FoxC1-mediated vascular niches and MSCs-induced angiogenesis jointly promote myocardial repair.

Supplementary Table SⅠ Primers for real-time RT-PCR of rat tissues.

Supplementary Table SII The antibodies for western blotting (WB), **enzyme-linked immunosorbent assays (ELISA)**, immunohistochemistry (IHC), flow cytometry (FCM), and immunofluorescence (IF).

Supplementary Figures and Tables

Figure S1

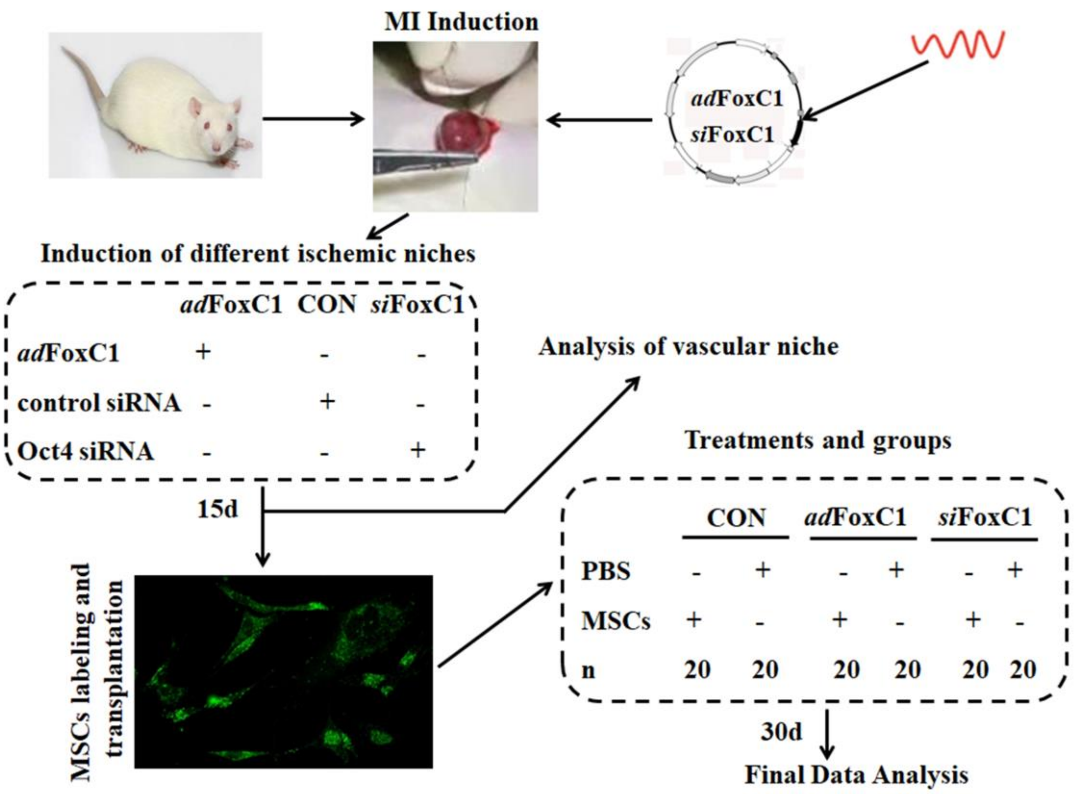

Figure S2

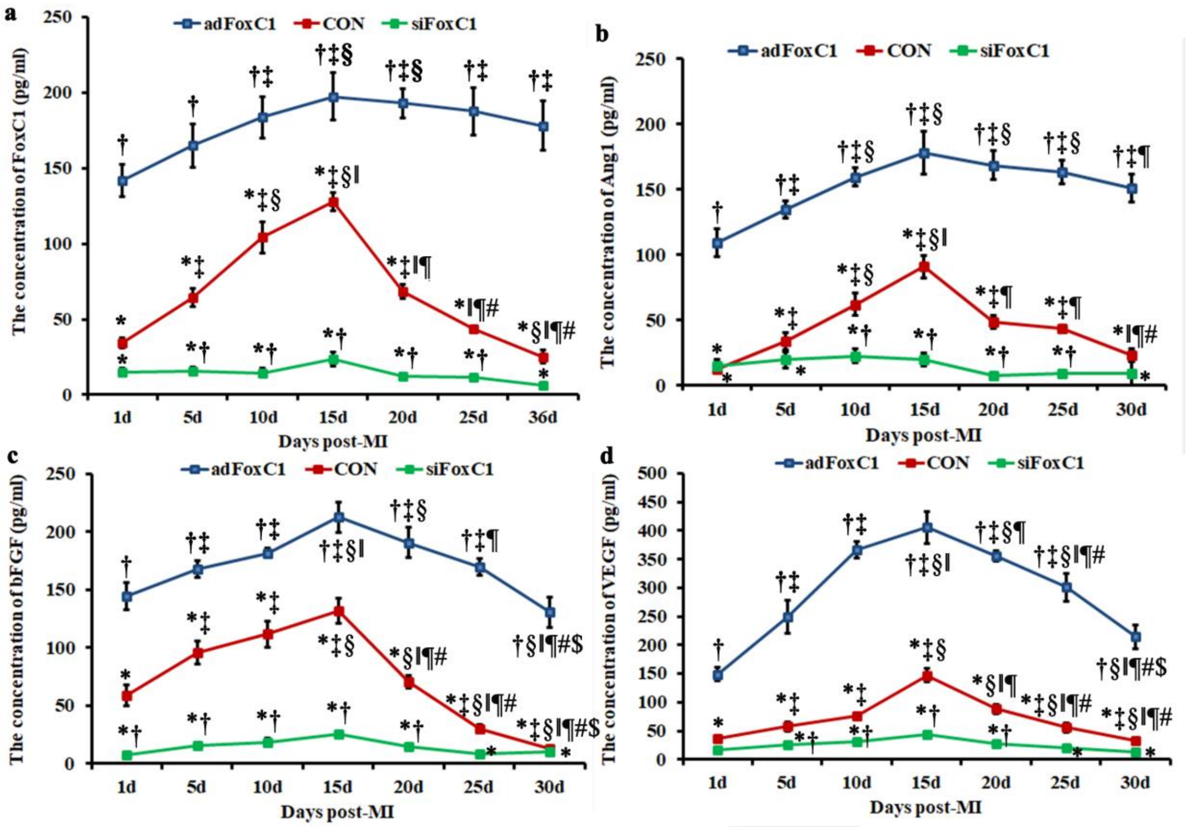
a
b
c
d
adFoxC1
CON
siFoxC1
The concentration of FoxC1 (pg/ml)
The concentration of Ang1 (pg/ml)
The concentration of bFGF (pg/ml)
The concentration of VEGF (pg/ml)
Days post-MI
1d
5d
10d
15d
20d
25d
36d
30d

Figure S3

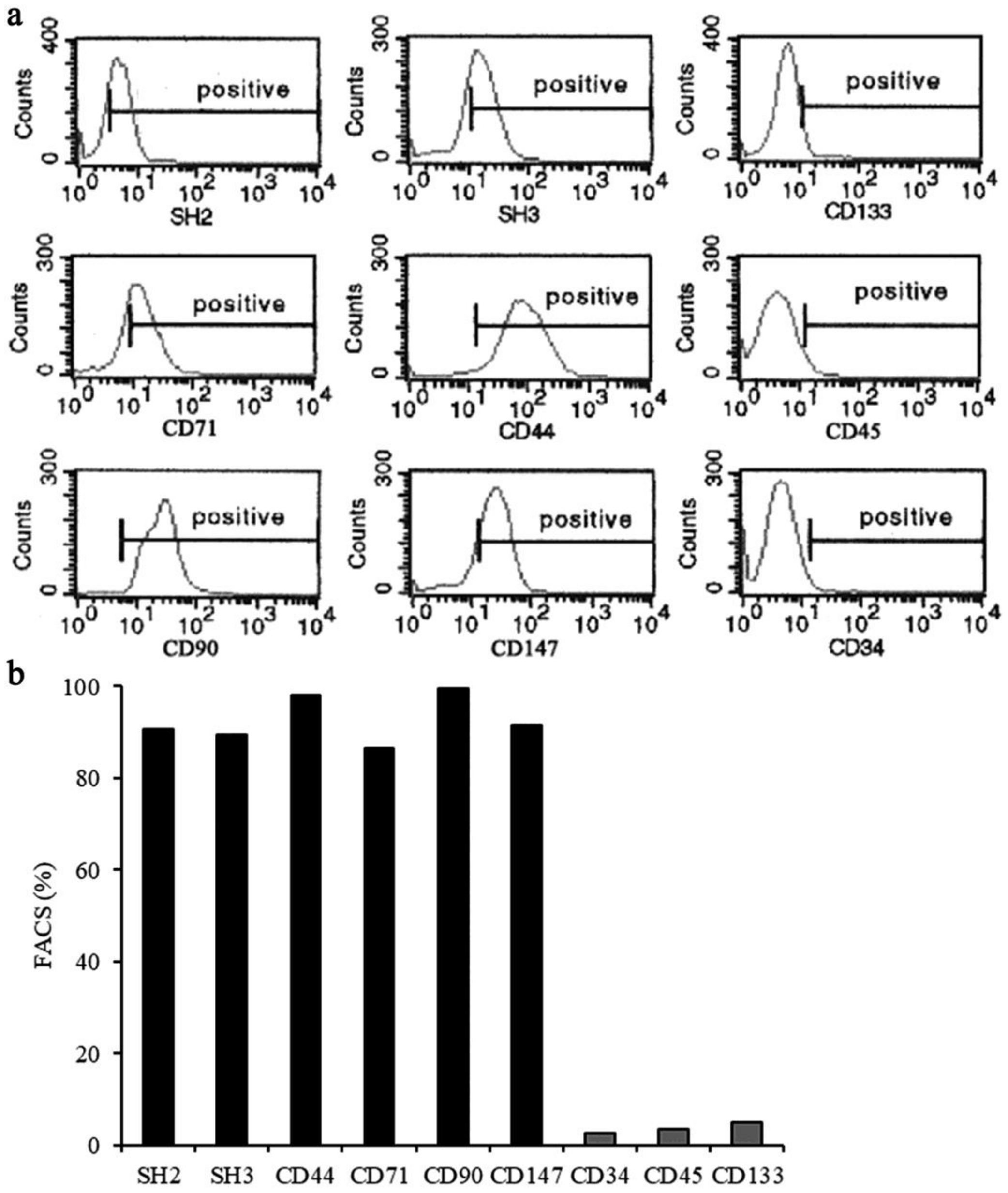
a
Counts
positive
SH2
SH3
CD133
CD71
CD44
CD45
CD90
CD147
CD34
b
FACS (%)
SH2 SH3 CD44 CD71 CD90 CD147 CD34 CD45 CD133

Figure S4

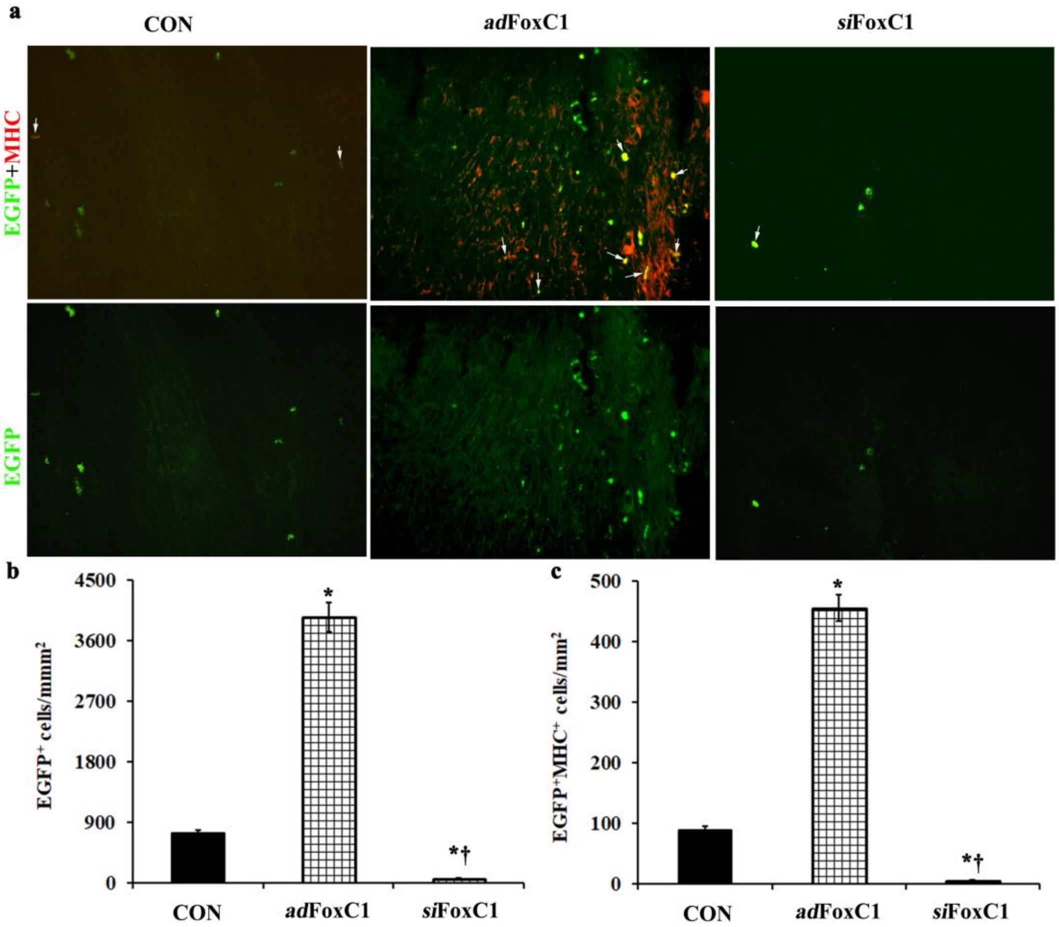
a
CON
adFoxC1
siFoxC1
EGFP+MHC
EGFP
b
4500
3600
2700
1800
900
0
EGFP+ cells/mmm2
*
*†
CON
adFoxC1
siFoxC1
c
500
400
300
200
100
0
EGFP+MHC+ cells/mm2
*
*†
CON
adFoxC1
siFoxC1

Figure S5

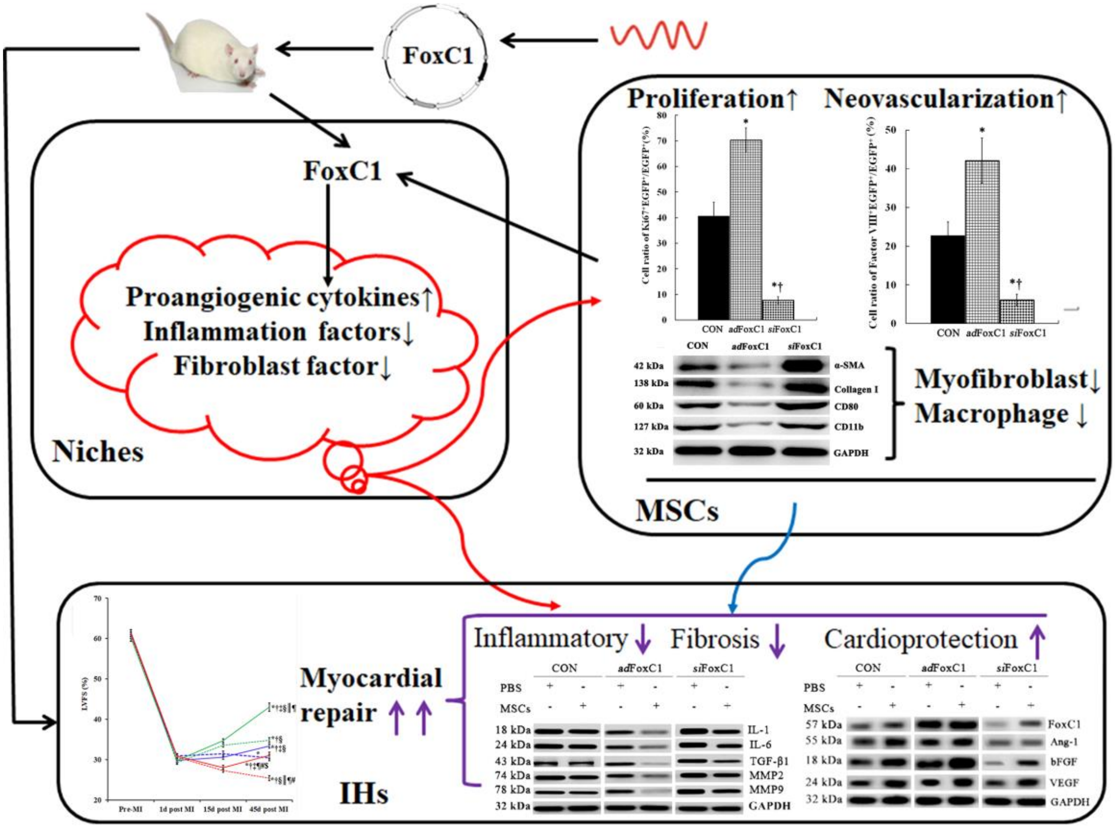

FoxC1
Proliferation↑
Neovascularization↑
FoxC1
Proangiogenic cytokines↑
Inflammation factors↓
Fibroblast factor↓
Niches
CON adFoxC1 siFoxC1
42 kDa α-SMA
138 kDa Collagen I
60 kDa CD80
127 kDa CD11b
32 kDa GAPDH
Myofibroblast↓
Macrophage↓
MSCs
Myocardial repair
Inflammatory
Fibrosis
Cardioprotection
PBS
MSCs
18 kDa IL-1
24 kDa IL-6
43 kDa TGF-β1
74 kDa MMP2
78 kDa MMP9
32 kDa GAPDH
57 kDa FoxC1
55 kDa Ang-1
18 kDa bFGF
24 kDa VEGF
32 kDa GAPDH
IHs
LVFS (%)
Pre-MI 1d post MI 15d post MI 45d post MI

Supplementary Tables

Table S 1 The primers for real-time RT-PCR

Gene	Description	Primer Sequence	Product size (bp)
FoxC1	Forkhead box C1	AAGACGGAGAACGGTACGTG<br>CGGGACTCTCGATTTTAGGCA	103
Ang-1	Angiopoietin 1	AACAGGAGGTTGGTGGTTTGATG<br>GAACATCCCCAGATTTATTTCAGGT	217
bFGF	basic fibroblast growth factor	CCATCAAGGGAGTGTGTGCG<br>CCCAGTTCGTTTCAGTGCCA	176
VEGF	Vascular endothelial growth factor	CCGACAGGGAAGACAATGGGA<br>GGGATGGGTTTGTCGTGTTTCT	149
IL-1	Interleukin-1	GCATGGCATGTGCTGAGTCT<br>ATGTCGGGCTGGTTCCACTA	90
IL-6	Interleukin-6	GCACCTCAGATTGTTGTTG<br>AGTGTCCTAACGCTCATAC	117
IL-4	Interleukin-4	CAGAAAAAGGGACTCCATGCACCG<br>TTGCGAAGCACCCTGGAAGCC	109
IL-10	Interleukin-10	TCCCAGTCAGCCAGACCCACA<br>AATCGATGACAGCGTCGCAGC	289
TGF-β1	Transforming growth factor beta 1	GACTACTACGCCAAGGAGGTC<br>GAGAGCAACACGGGTTCAG	149
MMP2	matrix metalloproteinase2	TTGACGGTAAGGACGGACTC<br>GGCGTTCCCATACTTCACAC	134
MMP9	matrix metalloproteinase9	AAGGGCGTCGTGGTTCCAACTC<br>AGCATTGCCGTCCTGGGTGTAG	210
GAPDH	Glyceraldehyde-3-phosphate dehydrogenase	CCGAGGGCCCACTAAAGG<br>TGCTGTTGAAGTCACAGGAGACA	67

Table S2 The antibodies for WB, IHC, FCM, and IF

Name	Company	Catalog	Molecular weight	Applications
SH2	LifeSpan BioSciences	LS-B7922		FCM, IHC, WB
SH3	Antibodies-online	ABIN5542542		FCM, IHC, WB, ELISA
CD44	LifeSpan BioSciences	LS-C13435		FCM, IHC, WB, ELISA
CD71	MyBioSource	MBS212212	70 kDa	FCM, IHC, WB, ELISA
CD90	MyBioSource	MBS213035	18 kDa	FCM, IHC, WB
CD147	MyBioSource	MBS249247	23 kDa	FCM, IHC, WB
CD34	MyBioSource	MBS520138		FCM, IHC
CD45	MyBioSource	MBS438123		FCM
CD133	MyBioSource	MBS462020	117 kDa	FCM, IHC, WB, ELISA
Ki67	Biorbyt	orb389335	310 kDa	IF, WB, IHC
FoxC1	Aviva	OASG02780	57 kDa	WB, IHC, IF
	Santa Cruz Biotechnology	ABIN3175115	57 kDa	ELISA, IF, IHC, WB
Ang1	Biorbyt	orb10091	55 kDa	WB, IHC, ELISA
bFGF	Bioss Inc.	bs-0217R	18 kDa	WB, IHC, IF
	MyBioSource	MBS551041	31 kDa	WB, IHC, ELISA
VEGF	Bioss Inc.	bs-0279R	24 kDa	WB, FCM, IF, IHC
	Biorbyt	orb100468	24 kDa	ELISA,IF,IHC-P
Factor VIII	Novus Biologicals	NB100-91761B	267 kDa	WB, IF, IHC
IL-1	Aviva	ARP54322_P050	18 kDa	IHC, WB
IL-6	BosterBio	A00102-2	24 kDa	IHC, WB
IL-4	Proteintech Group Inc	66142-1-Ig	18 kDa	WB, IHC, IF, ELISA
IL-10	Wuhan Fine Biotech	FNab04211	21 kDa	WB, IHC, ELISA
TGFβ1	Aviva	ARP37894_P050	43 kDa	IHC, WB, IF
MMP2	Aviva	AVARP20016_T100	74 kDa	IHC, WB
MMP9	Aviva	ARP33090_T100	78 kDa	IHC, WB
MHC	Bioss Inc.	bs-5885R	223 kDa	WB, IF, IHC
α-SMA	United States Biological	MBS9400363	42 kDa	WB, IF, IHC
	HuaBio	ET1607-43	42 kDa	WB, IHC, FCM
Collagen I	Abcam	ab84956	138 kDa	WB, IF, IHC
	Novus Biologicals	NBP2-46875	139 kDa	WB, IHC, FCM, ELISA
CD80	Biorbyt	orb13183	60 kDa	WB, IF, IHC
	MyBioSource	MBS5309376	33 kDa	FCM
CD11b	MyBioSource	MBS177463	127 kDa	WB, IF, IHC
	Novus Biologicals	NB110-89474	127 kDa	WB, IHC, FCM, IF
GAPDH	MyBioSource	MBS8243111	32 kDa	WB, IHC
